# Cultural Perspectives in Facial Allotransplantation

**Published:** 2012-08-23

**Authors:** Pearlie W.W. Tan, Ashish S. Patel, Peter J. Taub, Joshua A. Lampert, George Xipoleas, Gabriel F. Santiago, Lester Silver, Hemin O. Sheriff, Tsan-Shiun Lin, Rodney Cooter, Franco Diogo, Bruno Salazaard, Byung Jun Kim, Yoon Ho Lee, Rei Ogawa

**Affiliations:** ^a^Singapore General Hospital, Singapore; ^b^Guys and St. Thomas' Hospital, London, United Kingdom; ^c^Mt. Sinai Medical Center, New York; ^d^Derriford Hospital, Plymouth, United Kingdom; ^e^Chang Gung Memorial Hospital, Kaohsiung, Taiwan; ^f^Waverly House Plastic Surgery Centre, Adelaide, Australia; ^g^Federal University of Rio de Janeiro, Rio de Janeiro, Brazil; ^h^Timone Children's Hospital, Marseille, France; ^i^Seoul National University Hospital, Seoul, Korea; ^j^Nippon Medical School Hospital, Tokyo, Japan

## Abstract

Facial allotransplantation is a clinical reality, proposed to provide improved functional and aesthetic outcomes to conventional methods of facial reconstruction. Multidisciplinary efforts are needed in addressing not just the surgical and immunological issues but the psychological and sociological aspects as well. In view of this, an international survey was designed and conducted to demonstrate that attitudes toward facial allotransplantation are highly influenced by cultural background. Of all countries surveyed, France had the highest percentage of respondents willing to donate their faces (59%) and Iraq had the lowest (19%). A higher percentage of respondents were willing to accepting a face transplant (68%) than donate their face after death (41%). Countries with a dominant Western population show greater percentages of willingness to accept a face transplant, as they exhibit more positive variables, that is, (1) acceptance of plastic surgery for disfigurement and for cosmetic reasons and (2) awareness to the world's first face transplant. Countries with a dominant Western population also show greater percentages of willingness to donate their faces after death, as they exhibit more positive variables, that is, (1) positive attitude to organ donation by being an organ donor themselves, (2) acceptance of plastic surgery if disfigured, and (3) awareness to the world's first face transplant. Although religion was sometimes cited as a reason for not donating their faces, data analysis has shown religion not to be a strong associating factor to willingness to donate a face after death.

Hailed as potentially the most significant advance in facial reconstruction in the past 30 years, the world's first partial facial allotransplantation was performed in November 2005 in Amien, France. Since then, around 20 facial allotransplantations have been performed in the world. Facial allotransplantation is a clinical reality, proposed to provide improved functional and aesthetic outcomes to conventional methods of facial reconstruction. “Careful patient selection is critical to the final success of facial transplantation,”^1^ which calls for multidisciplinary efforts in addressing not just the surgical and immunological issues but the psychological and sociological aspects. This would be targeted not just to the recipient but to the donor as well.

In view of this, we have designed and conducted an international survey, and our purpose is to demonstrate that attitudes toward facial allotransplantation are highly influenced by cultural background.

## MATERIALS AND METHODS

A 10-item tick-box questionnaire was designed (Table 1) and authors from the United States, the United Kingdom, China, France, Turkey, Australia, Brazil, Iraq, Korea, Japan, Singapore, India, and Taiwan were contacted. These countries were selected to represent a spectrum of different cultures. Author contact details were obtained through a search of online journals relating to plastic and reconstructive surgery as we felt that there would be a higher chance this pool of responders would be interested in the subject matter. We did not receive any response from China, Turkey, and India.

The questionnaire was e-mailed and each author was asked to send the questionnaire to random samples of people, regardless of age, sex, religion, and educational qualifications. We received 2694 completed surveys: 505 from the United States (New York, Chicago, and Virginia), 349 from the United Kingdom (London), 315 from Iraq (Baghdad), 274 from Japan (Tokyo), 251 from Singapore, 249 from Brazil (Rio de Janeiro), 237 from Australia (Adelaide), 233 from Taiwan (Taipei), 167 from Korea (Seoul), and 114 from France (Marseille). The median age was 33 years, with 59% (n = 1590) female respondents and 41% (n = 1104) male respondents (Table 2).

The following independent variables were analyzed: (1) sociopersonal (age, religion, gender, and educational qualifications), (2) attitude to surgery (personal experience of having had surgery, (3) attitude to organ donation and awareness of transplantation (being an organ donor, having a relative with a transplanted kidney, attitude to possibility of requiring a organ transplant themselves), (4) attitude to plastic surgery (acceptance of plastic surgery for cosmetic enhancement vs plastic surgery only if disfigured), and (5) awareness of the first face transplant that took place in France.

Dependent variables studied were willingness to (1) accept a face transplant and/or (2) donate their face after death. A Multilogistic Regression Analysis was performed (using SPSS v14.0; SPSS Inc, Chicago, IL) to analyze the data. Multivariable-adjusted odds ratios (OR) were calculated with 95% confidence intervals (CI) to determine how the independent variables would influence respondents' willingness to accept a face transplant and/or donate their faces after death.

## RESULTS

Multinomial logistic regression analysis was performed to identify variables that increased the likelihood of respondents accepting a face transplant and donating their faces after death (Tables 3 and 4). A higher percentage of respondents were willing to accepting a face transplant (68%) than donate their faces after death (41%) (Table 3).

### Attitudes to accepting a face transplant

The strongest variables associated with a willingness to accept a face transplant (Table 4) were acceptance of plastic surgery if disfigured (OR = 2.79, 95% CI: 2.05-3.80, *P* < .01) and for cosmetic enhancement (OR = 2.08, 95% CI: 1.63-2.65, *P* < .01), acceptance of an organ transplant if needed (OR = 2.04, 95% CI: 1.60-2.60, *P* < .01), awareness of the first face transplant (OR = 1.91, 95% CI: 1.49-2.45, *P* < .01) and being a woman (OR = 1.42, 95% CI: 1.13-1.78, *P* < .01). With regard to religious beliefs, only Roman Catholics were less likely to accept a face than Christians (OR = 0.67, 95% CI: 0.46-0.96, *P* < .05). No association was found between education level and a willingness to accept a face transplant.

### Comparing countries

**Figure 1:**
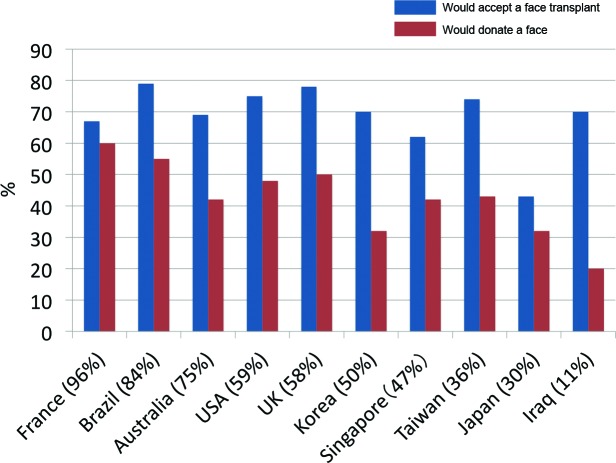
Awareness of the world's first partial face transplant and willingness to accept or donate a face for transplant. Comparing awareness of the world's first partial face transplant and willingness to accept a face transplant and willingness to donate their face after death.

Compared with French respondents, Japanese respondents were statistically much less likely to accept a face transplant (OR = 0.30, 95% CI: 0.13-0.73, *P* < .01) (Table 5). Forty-two percent of Japanese respondents were willing to accept a face transplant (vs 66% French respondents), and the most commonly cited reasons for not accepting were “personal,” with comments such as “ancestors would not forgive me” and “what is meant to be will be.” Other countries less likely than France to accept a face transplant are Australia, the United Kingdom, France, Iraq, Japan, Korea, Taiwan, and Singapore. Only the United States and Brazil had respondents more willing to accept a face transplant, but these results were not statistically significant. Awareness of the world's first face transplant was generally lower in the Asian countries surveyed, Japan (30%), Korea (50%), Taiwan (36%), and Singapore (47%), compared with the United States (59%), the United Kingdom (58%), Brazil (84%), Australia (75%), and France (96%), which correlates with generally lower rates of acceptance of a face transplant (Fig 1).

### Attitudes to donating a face after death

The strongest variables associated with a willingness to donate their face after death (Table 4) were carriage of an organ donor card (OR = 2.41, 95% CI: 1.85-3.15, *P* < .01), acceptance of plastic surgery if disfigured (OR = 1.98, 95% CI: 1.42-1.75, *P* < .01), acceptance of an organ transplant if needed (OR = 1.58, 95% CI: 1.26-1.99, *P* < .01), awareness of the first face transplant (OR = 1.89, 95% CI: 1.53-2.34, *P* < .01), and being a woman (OR = 1.29, 95% CI: 1.05-1.58, *P* = .01). With regard to religious beliefs, only those who responded as “no religion” (OR = 1.61, 95% CI: 1.10-2.53, *P* < .02) compared with Christians were more willing to donate their face.

### Comparing countries

**Figure 2:**
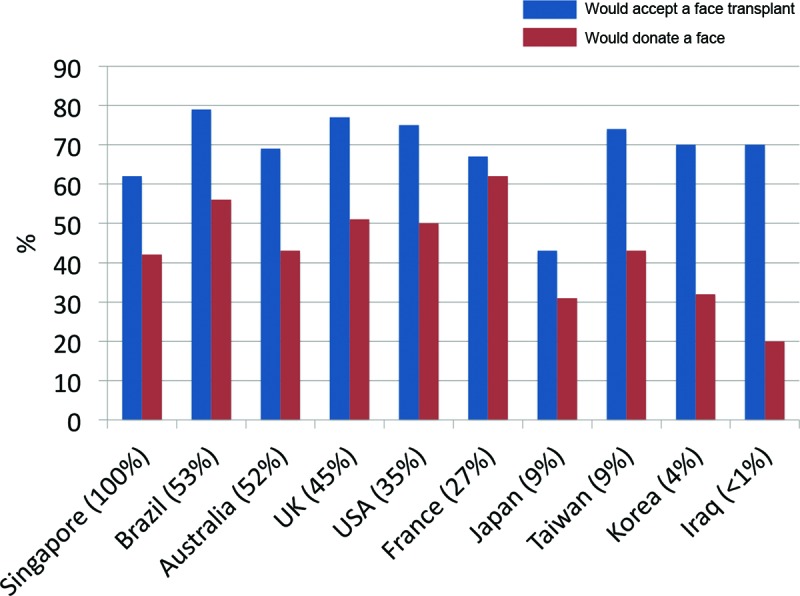
Organ donor card carriage in all countries surveyed and willingness to accept or donate a face for transplantation. Comparing organ donor card carriers in all countries surveyed and willingness to accept a face transplant and willingness to donate their face after death. Singapore is highlighted, among the respondents surveyed, many did not know that organ donation was an “opt out” system.^7^ Therefore, this result is an overestimate of the number of organ donor card carriers in Singapore. Respondents who did not “opt out” were default organ donors due to lack of knowledge of local policy versus having made a proactive choice to being an organ donor. The correlation between a high percentage of organ donors (positive variable to willingness to donate one's face) and low percentage of willingness to donate their face among Singaporeans should be regarded as a confounded correlation.

Respondents from France showed the highest percentage of willingness to donate their face after death (59%). Respondents from the United States (OR = 0.65, 95% CI: 0.33-1.27. p = 0.21), Iraq (OR = 0.26, 95% CI: 0.01-0.72, *P* = .01), Japan (OR = 0.84, 95% CI: 0.39-1.82, *P* = .67), Singapore (OR = 0.29, 95% CI: 0.13-0.64, *P* < .01), and Korea (OR = 0.28, 95% CI: 0.13-0.61, *P* < .01) were statistically much less likely to donate their face. Interesting comments were received from Singapore, “I don't want to ruin my body” and “ancestors will not recognize me when I go to heaven,” comments from Iraq, “against my religion,” comments from Japan “desecration of body would disturb my ancestors,” and from the United States “family would be very sad” and “don't want to lose my identity.” France showed the highest percentage of participants willing to donate their faces after death (59%). Correlating with the variables that positively influenced willingness to donate: 27% French respondents were organ donor card carriers, a high percentage of respondents would accept plastic surgery if disfigured (84%), and they ranked the highest in awareness to the first face transplant (96%), which was expected. Conversely, Iraq showed the lowest percentage of respondents willing to donate their faces after death (19%) where less than 1% of respondents were organ donor card carriers, a small percentage of respondents were willing to accept plastic surgery if disfigured (46%) and they also ranked the lowest in awareness of the world's first face transplant (11%). Across the Asian countries, there were lower percentages of organ donor card carriers—Japan (9%), Korea (9%), and Taiwan (4%), compared with the United States (35%), Brazil (53%), France (27%), the United Kingdom (45%), and Australia (52%), which correlates with their generally lower percentages compared with their Western counterparts in willingness to donate their faces (Fig 2). Although it appears that Singapore has the highest percentage of organ donor card carriers, the first author noted that many Singaporeans surveyed did not realize that organ donation was an “opt out” system.^2^ Therefore, this percentage of organ donor card carriers is an overestimate of the number of willing organ donors in Singapore. Respondents who did not “opt out” were default organ donors due to lack of knowledge of local policy versus having made a proactive choice to being an organ donor. Therefore, the high percentage of organ donors and low percentage of willingness to donate their faces among Singaporeans should be regarded as a confounded correlation. France also has an “opt out” policy for organ donation since 1994, and our coauthor (Salazard) made no mention of those surveyed not knowing this fact.

## DISCUSSION

Facial allotransplantation is a clinical reality that has been described by the Royal College of Surgeons as the inevitable next step in facial reconstruction.^3^ With societies becoming more multicultural, it would serve us well to better understand the impact of different cultural backgrounds to the acceptance of this procedure.

In this study, there were contrasting themes identified in countries where Eastern influence is dominant, that is, Taiwan, Singapore, Japan, and Korea, which are not mirrored in other countries where Western influence is dominant. Chinese respondents in Singapore and Taiwan mentioned beliefs that their body parts come from their parents and any removal of an organ is seen as disrespectful. They would be too embarrassed to meet their ancestors and be “unable to rest in peace.” Chinese and East Asians are greatly influenced by Confucianism, which has existed throughout East Asia since ancient times; it associates an intact body with respect for ancestors and nature.^4^ This may also explain why they have traditionally lower solid organ donation rates than their Western counterparts.^4^^,^^5^ Of the East Asian countries, the Japanese had the lowest rate of willingness to donate their faces (5%). Shinto, Buddhism, and Confucianism have long influenced Japanese culture, and there is a strong belief that a human being is the integration of body, mind, and spirit. Therefore, even after death a person remains as an integrated whole so removal of an organ is perceived as disturbing this integrated unit, a concept that one Japanese responder said “cannot (be) accept(ed) ethically.”

In contrast to Confucianism, Christianity plays a larger part in shaping Western culture.^6^ In Christianity, human beings are perceived as a synthesis of body and soul, but when someone dies, his or her soul ascends and what is left is no longer seen as a person. There is no emphasis of the soul being accountable for the body left behind. And, the donation of one's organs is seen as an act of love and generosity^7^ versus a desecration of one's body.

Of all countries surveyed, France had the highest percentage of respondents willing to donate their faces (59%) and Iraq had the lowest (19%). The percentage of people aware of the first face transplant was coincidentally highest and lowest in France and Iraq, respectively. Awareness is a strong associating factor to willingness to donate one's face after death. This result does not imply that raising awareness in one country will lead to a higher percentage willing to donate their face, but it does suggest that if surgeons decide to operate on a suitable candidate in countries where there is very little knowledge of the procedure, considerable attention should be paid in driving public awareness to reduce possible backlash from a relatively uninformed public.

Interestingly, even though willing to donate major organs, only 56% of organ donor card carriers were willing to donate their face after death, 26% responded “no” and 18% were unsure. This reflects the attitude to donating one's face conjures a wholly different response to donating one's liver or kidney.

In conclusion, a higher percentage of respondents were willing to accepting a face transplant (68%) than donate their face after death (41%). Countries with a dominant Western population show greater percentages of willingness to accept a face transplant, as they exhibit more positive variables, that is, (1) acceptance of plastic surgery for disfigurement and for cosmetic reasons and (2) awareness to the world's first face transplant. Countries with a dominant Western population also show greater percentages of willingness to donate their faces after death, as they exhibit more positive variables, that is, (1) positive attitude to organ donation by being an organ donor themselves, (2) acceptance of plastic surgery if disfigured, and (3) awareness to the world's first face transplant. Although religion was sometimes cited as a reason for not donating their face, data analysis has shown religion not to be a strong associating factor to willingness to donate a face after death.

## Figures and Tables

**Table 1:** Example questionnaire

1.	Age
2.	Religion (Buddhist/Christian/Catholic/Jewish/Taoist/Muslim/Hindu/Others/None)
3.	Gender
4.	Educational qualifications (primary/secondary school/tertiary level/university/postgraduate)
5.	Have you ever had surgery? (Yes/No)
6.	Do you carry an organ donor card? (Yes/No)
7.	Have you or a family member ever received an organ transplant? (Yes/No)
	If no; and if you required an organ transplant, for example, kidney that comes with life-long medication with many side effects, would you still accept it? (Yes/No)
	If No; because of (a) cost, (b) I will never want a transplanted organ, (c) I do not want the side effects of medication.
8.	Would you consider plastic surgery for: (a) Cosmetic reasons? (Yes/No)
	(b) If you were disfigured? (Yes/No)
9.	Recently a woman in France received a face transplant, are you aware of this event? (Yes/No)
10.	Would you consider a face transplant if you were severely disfigured after an accident? (Yes/No/Not sure)
	If no; because of (a) religious beliefs, (b) personal feelings, (c) I feel it is unsafe, (d) cost.
11.	Would you consider donating your face afterlife to someone who needs a face transplant? (Yes/No/Not sure)
	If no; because of (a) religious beliefs, (b) personal feelings, (c) I feel It is unsafe.

**Table 2:** Demographics

Age, y		Education level	
Mean	37	Primary	3.9% (n = 105)
Median	33	Secondary	12.7% (n = 341)
Range	14-88	Tertiary	23.9% (n = 644)
		University	46.7% (n = 1257)
Sex (women:men)	59%:41% (1590:1104)	Postgraduate	12.8% (n = 346)
Country (%, n = 2694)		Religion	
United States	18.7% (n = 505)	Christian	24.1% (n = 648)
United Kingdom	13.0% (n = 349)	Catholic	21.7% (n = 585)
Iraq	11.7% (n = 315)	None	9.3% (n = 250)
Japan	10.2% (n = 274)	Other	15.1% (n = 407)
Singapore	9.3% (n = 251)	Buddhist	11.7% (n = 314)
Brazil	9.2% (n = 249)	Jewish	2.9% (n = 79)
Australia	8.8% (n = 237)	Hindu	1.7% (n = 47)
Taiwan	8.6% (n = 233)	Muslim	13.5% (n = 364)
Korea	6.2% (n = 167)		
France	4.2% (n = 114)		

**Table 3:** Willingness to accept a face transplant or donate a face for transplantation (n = 2694)

	Accept	Donate
Yes	68% (n = 1817)	41% (n = 1096)
No	18% (n = 491)	36% (n = 973)
Not Sure	14% (n = 386)	23% (n = 625)

**Table 4:** Characteristics associated with willingness to accept a face transplant or donate a face for transplantation*

	Willingness to Accept a Face Transplant		Willingness to Donate a Face After Death	
Characteristic	Odds Ratio (95% CI)	*P*	Odds Ratio (95% CI)	*P*
Age	1.00 (1.00-1.01)	.26	**1.01 (1.00-1.02)**	**.04**
Sex				
Men	1.00 (reference)		1.00 (reference)	
Women	**1.42 (1.13-1.78)**	**<.01**	**1.75 (1.39-2.21)**	**<.01**
Country				
France	1.00 (reference)		1.00 (reference)	
United States	1.05 (0.48-2.32)	.90	**0.65 (0.33-1.27)**	**.21**
United Kingdom	0.76 (0.34-1.70)	.50	0.80 (0.39-1.62)	.53
Iraq	0.72 (0.23-2.21)	.72	**0.26 (0.01-0.72)**	**.01**
Japan	**0.30 (0.13-0.72)**	**<.01**	**0.84 (0.39-1.82)**	**.67**
Singapore	0.43 (0.18-1.02)	.43	**0.29 (0.13-0.64)**	**<.01**
Brazil	1.90 (0.74-4.87)	.18	1.82 (0.85-3.8)	.12
Australia	0.49 (0.21-1.14)	.10	0.53 (0.25-1.11)	.09
Taiwan	0.85 (0.35-2.05)	.71	0.52 (0.24-1.14)	.10
Korea	0.75 (0.31-1.83)	.53	**0.28 (0.13-0.61)**	**<.01**
Education				
Primary school	1.00 (reference)		1.00 (reference)	
Secondary school	0.73 (0.38-1.42)	.36	**3.55 (1.46-8.67)**	**<.01**
Tertiary education	0.87 (0.48-1.60)	.66	**3.71 (1.57-8.80)**	**<.01**
University degree	0.86 (0.48-1.56)	.62	**4.83 (2.08-11.26)**	**<.01**
Postgraduate degree	0.95 (0.49-1.84)	.87	**6.04 (2.49-14.65)**	**<.01**
Religion				
Christian	1.00 (reference)		1.00 (reference)	
Catholic	**0.67 (0.46-0.96)**	**.03**	0.78 (0.56-1.10)	.15
Buddhist	1.35 (0.86-2.13)	.20	0.97 (0.61-1.54)	.90
Jewish	0.94 (0.42-2.08)	.87	**0.45 (0.23-0.90)**	**.02**
Muslim	1.40 (0.60-3.25)	.44	0.51 (0.23-1.13)	.10
Hindu	0.56 (0.25-1.24)	.15	0.76 (0.33-1.75)	.52
Others	0.77 (0.51-1.17)	.22	0.71 (0.47-1.05)	.90
No religion	1.62 (1.00-2.63)	.05	**1.24 (0.80-1.92)**	**.34**
Organ donor card status				
Noncarriers	1.00 (reference)		1.00 (reference)	
Carriers	0.90 (0.65-1.25)	<.53	0.86 (0.63-1.17)	.34
Previous surgery				
No	1.00 (reference)		1.00 (reference)	
Yes	1.08 (0.84-1.38)	.55	1.06 (0.83-1.35)	.64
Would have cosmetic surgery				
No	1.00 (reference)		1.00 (reference)	
Yes	**2.08 (1.63-2.65)**	<.01	0.92 (0.73-1.16)	.47
Would have surgery if disfigured				
No	1.00 (reference)		1.00 (reference)	
Yes	**2.79 (2.05-3.80)**	<.01	1.01 (0.72-1.43)	.93
Experience of organ transplant				
No family member with transplant	1.00 (reference)		1.00 (reference)	
Family member with transplant	0.68 (0.41-1.13)	.14	1.01 (0.60-1.70)	.97
Would accept organ transplant				
No	1.00 (reference)		1.00 (reference)	
Yes	**2.04 (1.60-2.60)**	**<.01**	**1.45 (1.13-1.88)**	<.01
Awareness of first face transplant				
Not aware	1.00 (reference)		1.00 (reference)	
Aware	**1.91 (1.49-2.45)**	**<.01**	1.20 (0.95-1.53)	.13

CI indicates confidence interval.

*Values in bold indicate a statistical significance.

**Table 5:** Number of participants from each country surveyed who are organ donor card carriers, are aware of the world's first face transplant, are willing to have plastic surgery for cosmetic reasons or if disfigured, and are willing to accept a face transplant or donate their face for transplantation

		*Willing to Have Plastic Surgery*			*Attitudes to Face Transplantation*	
	Organ Donor Card Carriers,* %	For Cosmetic Reasons, %	If Disfigured, %	Awareness of World's First Transplant, %	Willing to Accept a Face, %	Willing to Donate a face, %
United States	35 (n = 179)	22 (n = 112)	94 (n = 477)	59 (n = 299)	73 (n = 367)	47 (n = 236)
(n = 505)						
United Kingdom	45 (n = 117)	21 (n = 72)	95 (n = 330)	58 (n = 201)	76 (n = 264)	49 (n = 172)
(n = 349)						
France	27 (n = 31)	17 (n = 19)	84 (n = 96)	96 (n = 110)	66 (n = 75)	59 (n = 67)
(n = 114)						
Australia	52 (n = 123)	26 (n = 62)	98 (n = 232)	75 (n = 177)	68 (n = 161)	42 (n = 99)
(n = 237)						
Brazil	53 (n = 132)	41 (n = 101)	98 (n = 244)	84 (n = 208)	77 (n = 192)	54 (n = 134)
(n = 249)						
Singapore	100 (n = 250)	28 (n = 71)	82 (n = 206)	47 (n = 118)	61 (n = 153)	41 (n = 103)
(n = 250)						
Japan	9 (n = 24)	4 (n = 10)	91 (n = 248)	30 (n = 81)	42 (n = 116)	30 (n = 81)
(n = 274)						
Taiwan	9 (n = 20)	2 (n = 4)	92 (n = 205)	36 (n = 81)	72 (n = 161)	42 (n = 93)
(n = 223)						
Korea	4 (n = 6)	0	86 (n = 143)	50 (n = 83)	68 (n = 114)	31 (n = 52)
(n = 167)						
Iraq	<1 (n = 1)	0	46 (n = 146)	11 (n = 34)	68 (n = 214)	19 (n = 59)
(n = 315)						

*In Singapore, policy on organ donation under HOTA (Human Organ Transplant Act) is an “opt-out” system. The lead author noted that many Singaporeans surveyed did not realize that organ donation is an “opt out” system. Therefore, this result is an overestimate of the number of organ donor card carriers in Singapore.
